# A facile and green procedure in preparing dibenzo-chromeno-phenazine-diones using an effectual and recyclable Brønsted acidic ionic liquid

**DOI:** 10.1038/s41598-024-73257-3

**Published:** 2024-11-05

**Authors:** Chou-Yi Hsu, Riyadh Abdulkareem, Harikumar Pallathadka, Vikrant Abbot, Mamata Chahar, Dilsora Abduvalieva, Yasser Fakri Mustafa, Usama S. Altimari, Abeer mhussan jabbar, Ahmed Hussein Zwamel

**Affiliations:** 1 Thunderbird School of Global Management, Arizona State University Tempe Campus, Phoenix, AZ 85004 USA; 2 Chemical, Biological and Radiological Safety Security Section, University of Anbar, Al-Anbar, Iraq; 3 Manipur International University, Imphal, Manipur India; 4 Chandigarh Group of colleges, jhanjeri, Chandigarh pharmacy college, 140307 Mohali, punjab India; 5 Department of Chemistry, NIMS University, Jaipur, India; 6 Department of Mathematics and Information Technologies, Tashkent State Pedagogical University, Bunyodkor avenue, 27, Tashkent, 100070 Uzbekistan; 7 Department of Pharmaceutical Chemistry, College of Pharmacy, University of Mosul, Mosul, 41001 Iraq; 8 Department of Medical Laboratories Technology, AL-Nisour University College, Baghdad, Iraq; 9 College of Pharmacy, National University of Science and Technology, Dhi Qar, Iraq; 10 Medical Laboratory Technique College, The Islamic University, Najaf, Iraq; 11 Medical Laboratory Technique College, The Islamic University of Al Diwaniyah, Al Diwaniyah, Iraq; 12 Medical Laboratory Technique College, The Islamic University of Babylon, Babylon, Iraq

**Keywords:** Dibenzo-chromeno-phenazine-diones. Multicomponent domino reactions (MCDRs). 1,3-n-propyl-bipyridinium bisulfonic acid-ditrifluoroacetate (PBPBSDT). Solvent-free medium, Catalysis, Medicinal chemistry, Organic chemistry

## Abstract

First, a Brønsted acid ionic liquid (BAIL) in the role of a double acid-base called 1,3-n-propyl-bipyridinium bisulfonic acid-ditrifluoroacetate (PBPBSDT) was produced, and its skeleton was determined via TGA, mass, ^13^C NMR, ^19^F NMR, ^1^H NMR and FT-IR data. Further, it was successfully applied in the preparation of dibenzo-chromeno-phenazine-dione derivatives (**1a**-**12a**, 9–15 min, 90–98%) via one-pot multicomponent domino reaction among 2 mmol 2-hydroxynaphthalene-1,4-dione, 1 mmol benzene-1,2-diamine, and 1 mmol aldehydes under optimal conditions (5 mol% of PBPBSDT, solvent-free, 60 °C). The proper reproducibility of the PBPBSDT homogeneous catalyst (5 times), solvent-free medium, reasonable TON (Turnover Number) and TOF (Turnover Frequency) numbers, the non-metallic framework of the catalyst, and the formation of C-N, C = N, C-C, C = C, C-O bonds in a single operation are the distinct advantages of this protocol.

## Introduction

Ionic liquids (ILs) are weakly coordinated ionic compounds (organic salts) that lead to the liquefaction of these materials below 100 °C or even at ambient temperature^1,2^. Using ILs in organic transformations as eco-friendly catalysts is deemed an advanced technology due to their outstanding properties such as easy recyclability, low vapor pressure, functional variability, non-flammability, acidity/basicity of cations/anions, less corrosion compared to mineral bases and acids, high thermal and chemical durability, tunable solubility, and capability to catalyze various synthetic pathways^3–6^. Brønsted acidic ionic liquids (BAILs) are an important subgroup of these liquids, which are widely used as efficient catalysts in organic synthesis due to their advantages of traditional liquid and solid acids, mild acidic nature, easy design and production, and good efficacy and selectivity^6,7^. Recently, some species of this subset whose cationic part contains SO_3_H bonded to positive nitrogen and their anionic part comprises basic sites (although weak like AlCl_4_ˉ) as a bi-functional catalyst (or dual acid-base) in various synthetic transformations have been reported in the literature^8–10^. Therefore, the synthesis of such ILs can be crucial in aiding reactions that simultaneously require acidic and basic sites to be catalyzed^11^.

Today, the development of simple, cost-effective, and green chemistry-friendly organic transformations has been of great significance in terms of environmental sustainability^12,13^. A fundamental step in this direction is using one-pot multicomponent domino reactions (MCDRs) under solvent-free conditions^14^. These reactions have provided the most efficient platform for the synthesis of complex compound libraries in the screening of active pharmaceutical and biological candidates^15–18^. MCDRs, at the same time as operating simplicity and improving atomic economy, can prevent costly and time-consuming procedures for the purification of diverse precursors; so they are a constant challenge in the front line of organic synthesis, especially in the generation of heterocyclic compounds^15–19^.

In the pharmaceutical industry, heterocyclic systems with chromene and phenazine segments (e.g., dibenzo-chromeno-phenazine-diones) are important because they are the vital framework of many drugs and biologically active substances^20–22^. Various biological properties of chromen core have been proven, including antimicrobial, antitumor, antifungal, antiasthmatic, antioxidant, and anti-HIV activities^23–28^. Also, phenazine systems present in natural and synthetic products exhibit various biological functions including antimicrobial, antibiotic, antiparasitic, antimalarial and antitumor activities in solid and leukemia tumors^29–33^. For example, pyridazinophenazinedione derivatives and pyridophenazinediones are well known for their anticancer properties^31,32^. However, notwithstanding their established biological applications, only very few protocols describing their production have been reported in the literature^34–38^. Therefore, it is very important to introduce new methods that allow the rapid and easy production of these valuable compounds while minimizing the disadvantages of previous methods.

Taking into account the above, by gathering the advantages of domino reactions, multicomponent reactions, bi-functional catalysts, solvent-free environment, and recyclable ILs, we presented a simple and effective protocol at a mild temperature of 60 °C for the synthesis of dibenzo-chromeno-phenazine-dione derivatives using PBPBSDT ionic liquid via one-pot MCDR of 2 mmol 2-hydroxynaphthalene-1,4-dione, 1 mmol benzene-1,2-diamine, and 1 mmol aldehydes. Notably, according to our investigations, this is the first report of the production of dibenzochromeno-phenazine-dione derivatives with an ionic liquid catalyst.

## Experimental

### Materials and apparatus

The description of the specifications of all starting materials and apparatus is given in the related files.

### Procedure for the preparation of ionic liquid PBPBSDT

**Fig. 1:**
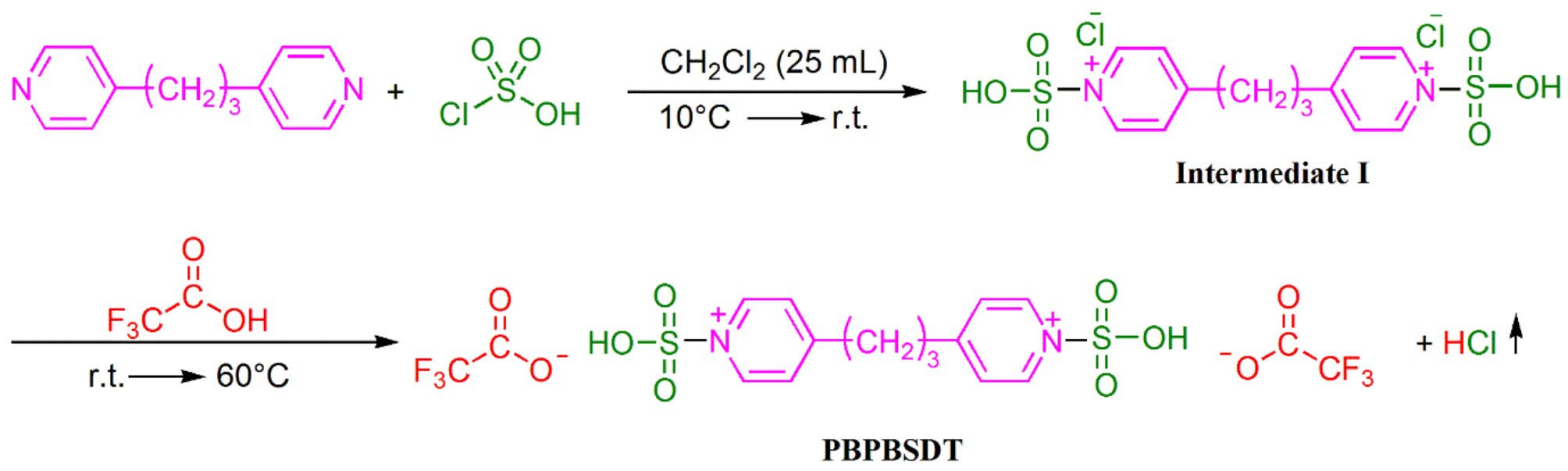
The preparation of PBPBSDT.

At the beginning, 4 mmol of 1,3-di(pyridin-4-yl)propane (dissolved in 25 mL dichloromethane) was gradually added to 8 mmol of chlorosulfuric acid (dissolved in 25 mL dichloromethane) within 5 min at 10 °C. Next, the resulting solution was permitted to heat to 25 °C, and it stirred at this temperature for 120 min. The dichloromethane was evaporated, and the resulting was cleaned with petroleum ether (3 × 4 mL), and it dried under vacuum at 70 °C to provide Intermediate **I**. At the last stage, 8 mmol of trifluoroacetic acid (TFA) was gradually added to 4 mmol of intermediate **I**. The resulting solution was stirred for 5 min at 25 °C, and then 60 min at 60 °C to synthesize PBPBSDT (a viscous yellow oil in 98% yield) (Fig. 1).

### General procedure for producing dibenzo-chromeno-phenazine-diones

**Fig. 2:**
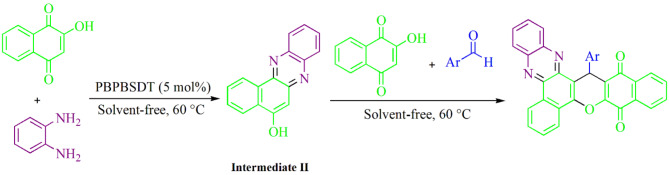
General procedure for producing dibenzo-chromeno-phenazine-diones.

In the first step under a domino reaction at 60 °C, a mixture of 5 mol% PBPBSDT, 1 mmol benzene-1,2-diamine, and 1 mmol 2-hydroxynaphthalene-1,4-dione was stirred in solvent-free conditions (5 min) to give Intermediate **II** (Fig. 2). Subsequently, during a multicomponent reaction, 1 mmol 2-hydroxynaphthalene-1,4-dione, and 1 mmol aldehyde were added to the Intermediate **II**. This mixture was stirred with TLC monitoring until the end of the reaction. After completely consuming the starting material, the residue was cooled to ambient temperature, and PBPBSDT was isolated (based on the recycle section technique). Eventually, the pure dibenzo-chromeno-phenazine-diones were achieved via recrystallization of the residue in 95% ethanol.

## Results and discussion

### Studies to confirm the structure of 1,3-n-propyl-bipyridinium bisulfonic acid-ditrifluoroacetate (PBPBSDT)

In this section, the structure of PBPBSDT was evaluated using Fourier transform infrared spectroscopy (FT-IR), fluorine (^19^F), carbon (^13^C), proton (^1^H) NMR, thermal analysis (TGA), and mass spectrometry.

**Fig. 3:**
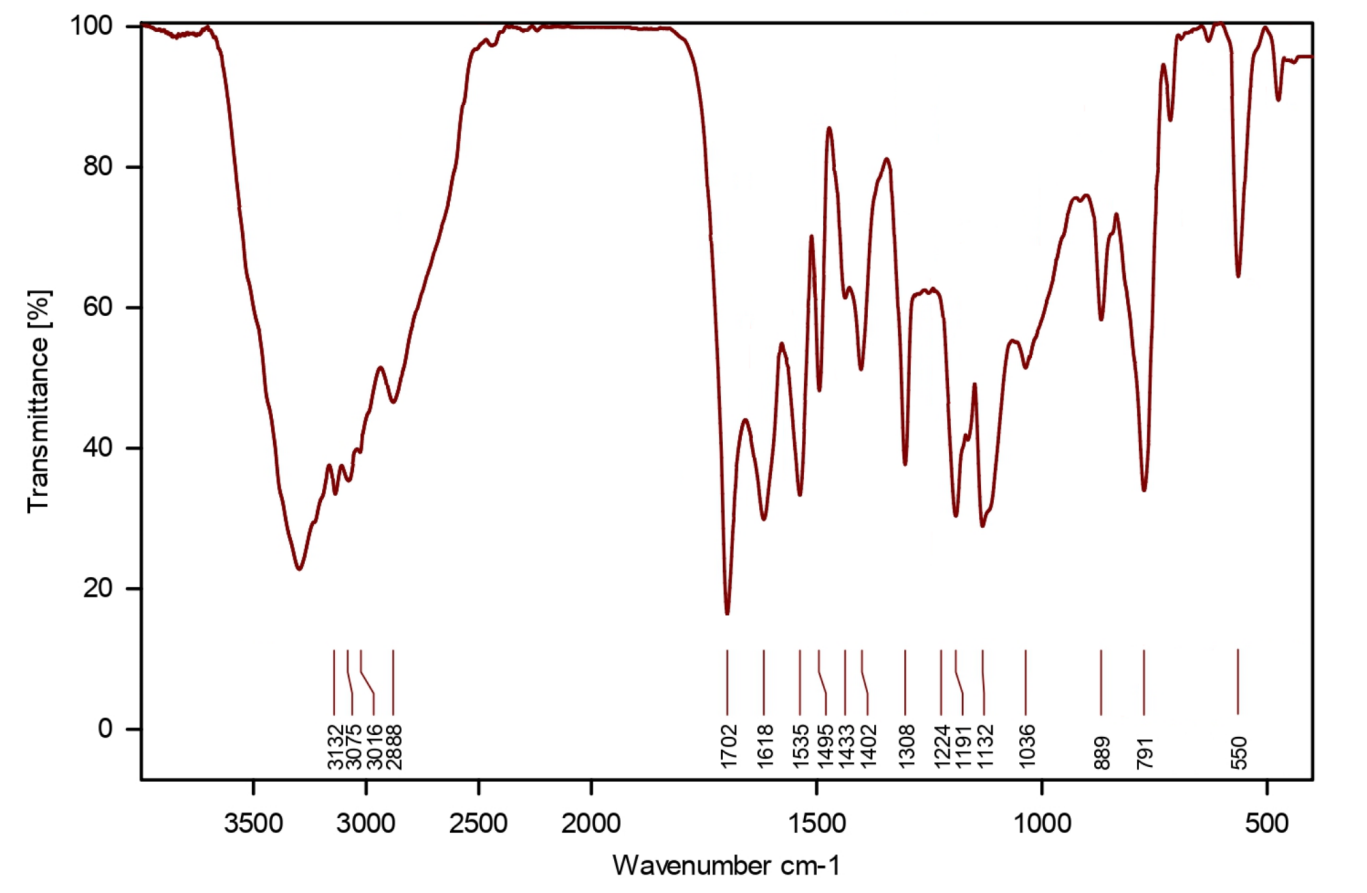
The FT-IR spectrum of PBPBS DT.

In Fig. 3, the FT-IR pattern of PBPBSDT is displayed, and the findings are compiled in Table 1. As can be observed in Table 1; Fig. 3, the wavenumbers corresponding to anticipated bonds and functional groups have appeared in the PBPBSDT spectrum. The literature supports our results^10,40–42^.

**Table 1:** The FT-IR data of PBPBSDT.

Wavenumber (cm^− 1^)	Related functional group or bond
2500–3500	O–H stretching vibration of SO_3_H
3132	C–H symmetric stretching =
3016	C–H symmetric stretching
1702	C = O stretching
1618	C = N stretching
1535, 1495	C = C stretching
1433	CH_2_ bending
1308	C–O stretching
1224	C–F stretching
1191 and 1132	S–O asymmetric and symmetric stretching
1036	S–OH bending
889	N–S stretching
791	C–H out-of-plane =
550	S–O bending

**Fig. 4:**
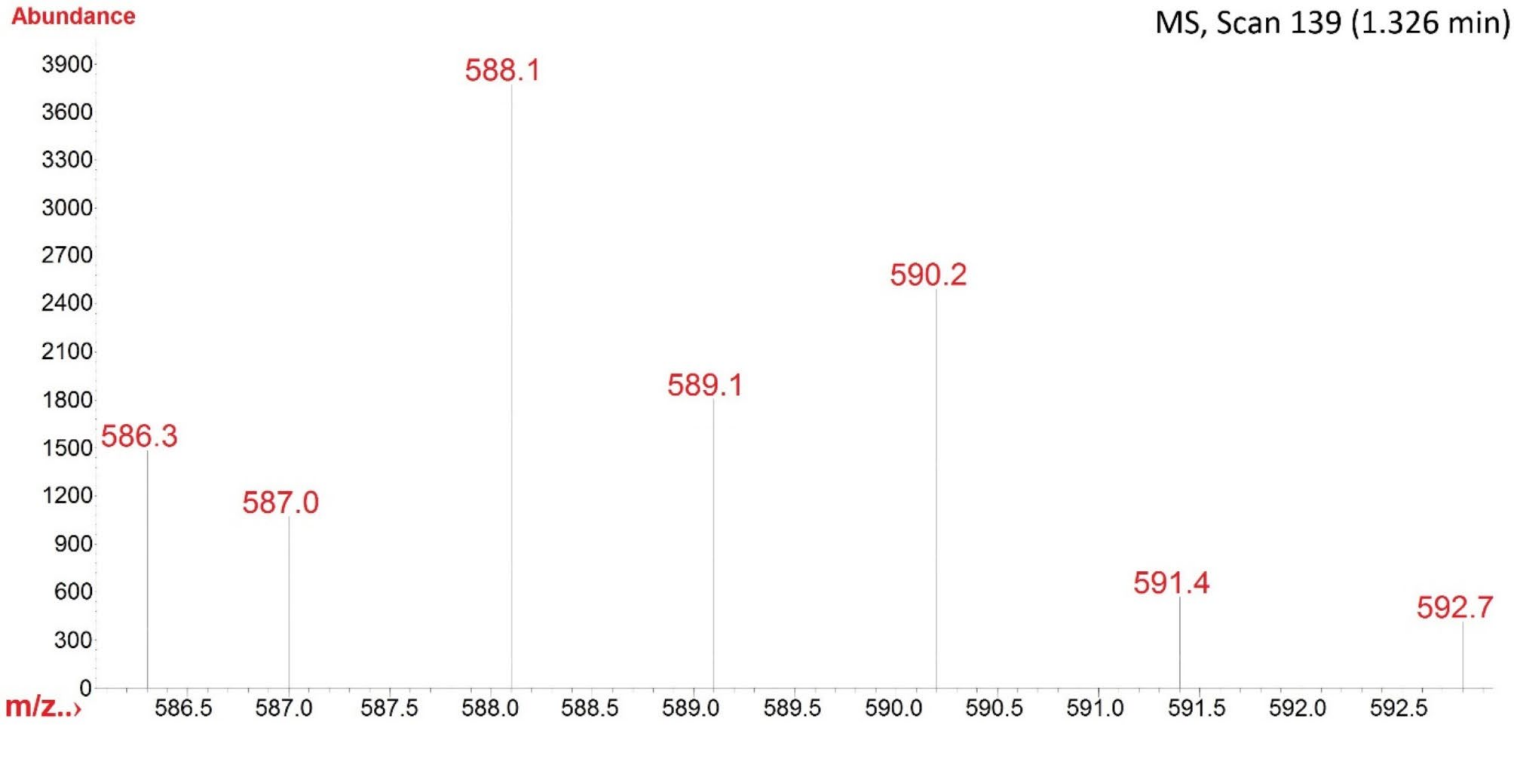
Part of the PBPBSDT mass spectrum pattern.

The mass spectrum of 1,3-n-propyl-bipyridinium bisulfonic acid-ditrifluoroacetate revealed the molecular mass (M^+^), (M^+^+1), and (M^+^+2) at *m/z* 588, 589 and 590, respectively (Fig. 4).

**Fig. 5:**
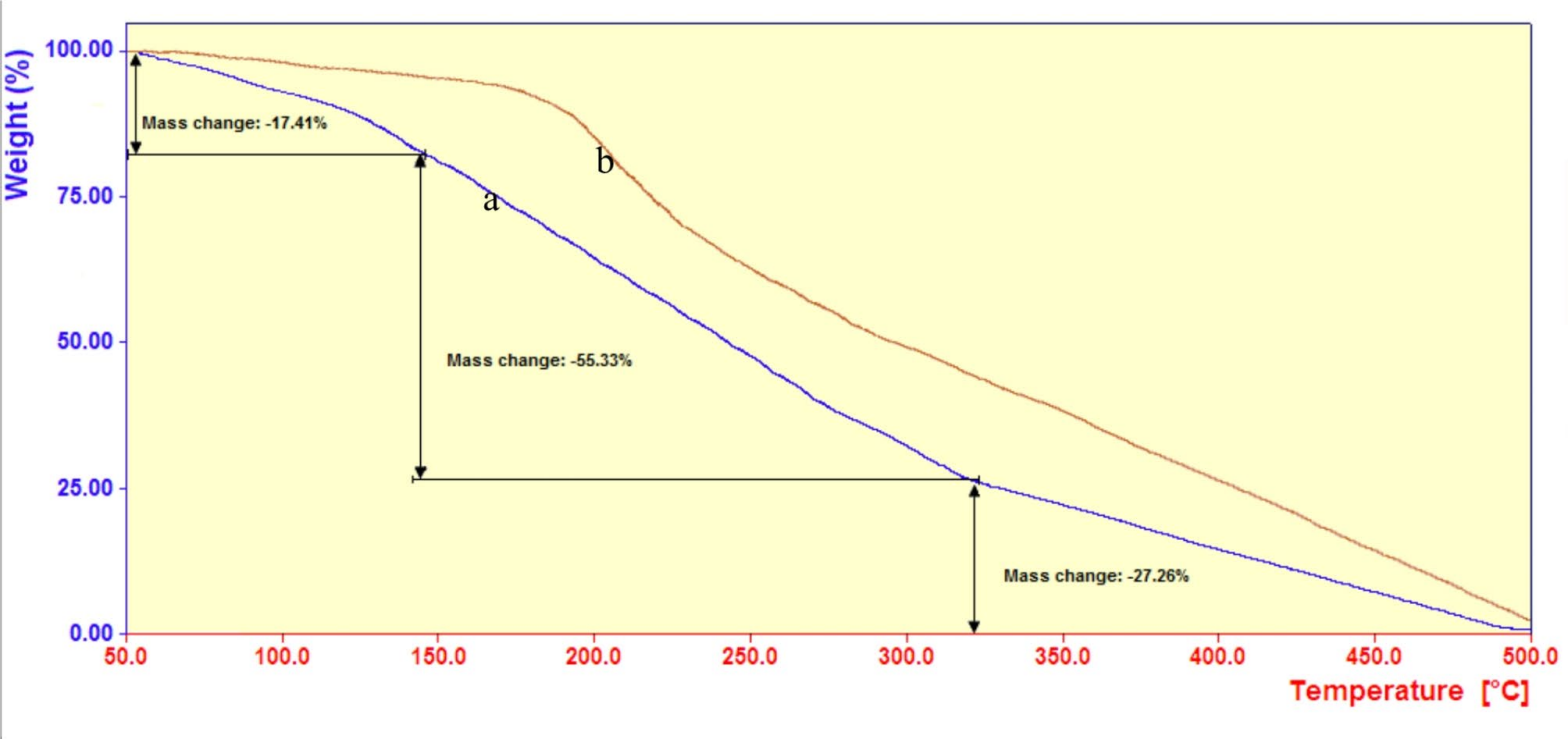
TGA diagrams of PBPBSDT (**a**), and intermediate **I** (**b**).

In the scope of 50–500 °C, the PBPBSDT thermal behavior was investigated by analyzing its TGA diagram (Fig. 5a). The results indicated that the weight loss of ionic liquid occurs in the following three temperature ranges: 50 to ~ 150 (− 17.41%), 150 to 322 °C (− 55.33%), and 322 to 500 °C (− 27.26%). Based on similar literature, the weight loss in the first stage can be attributed to the evaporation of the absorbed solvents in PBPBSDT, and in the second and third stages to the decomposition of its organic framework^8^. Therefore, according to the onset temperature of PBPBSDT weight loss (i.e., from 150 °C onwards), it can be said that this ionic liquid has enough stability to catalyze an MCDR at a mild temperature of 60 °C. In another study, the thermal stability of intermediate **I** (Fig. 5b), was compared with PBPBSDT under the same conditions. The results showed that the thermal behavior of these two ionic liquids is similar, but decomposition onset temperature of intermediate **I** is slightly higher (50 °C). This is due to the thermal stability of chloride anion (Clˉ) compared to trifluoroacetate (CF_3_COOˉ). These results are consistent with previously observed trends in ionic liquids^43^.

**Fig. 6:**
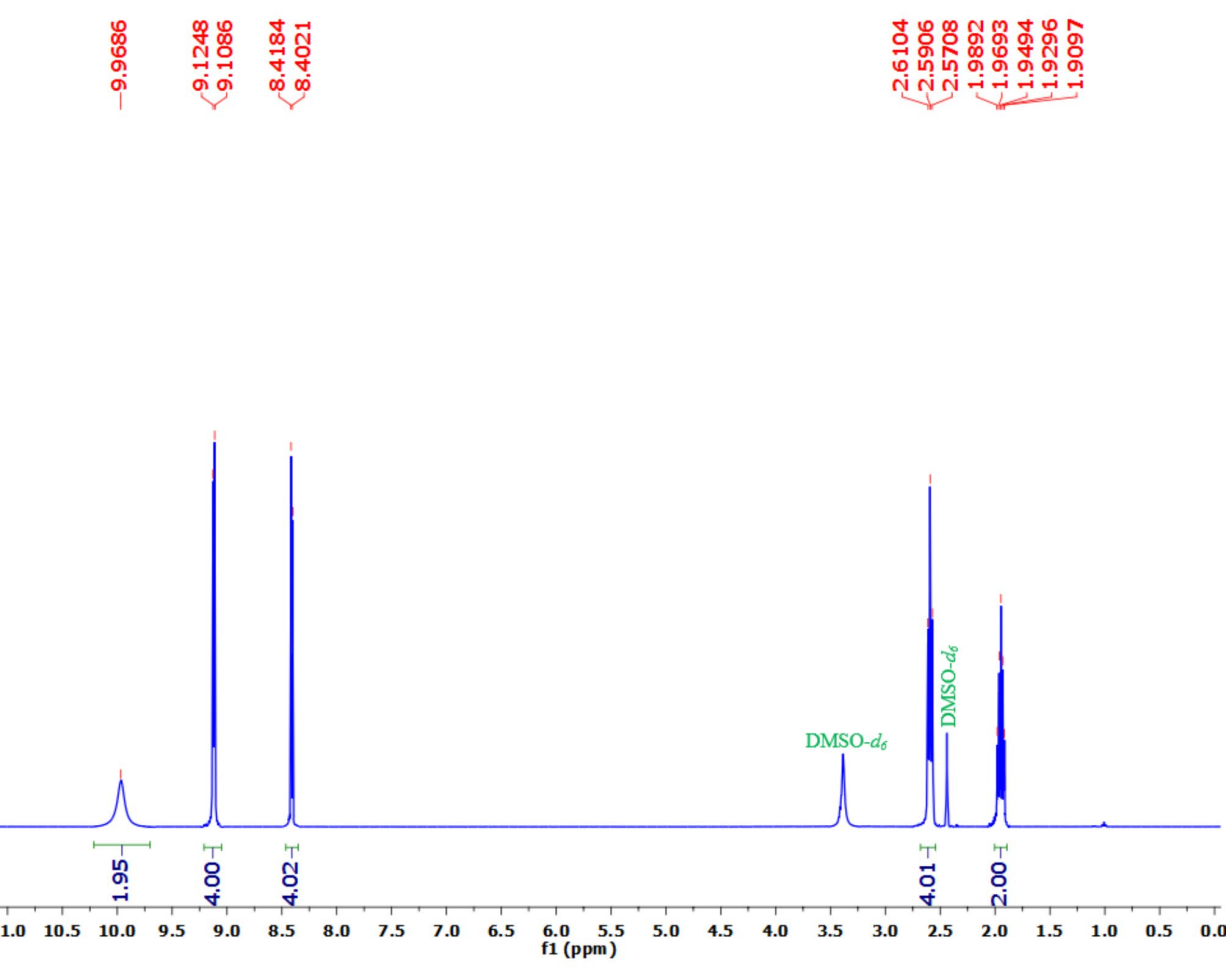
^1^H NMR spectrum of PBPBSDT.

**Fig. 7:**
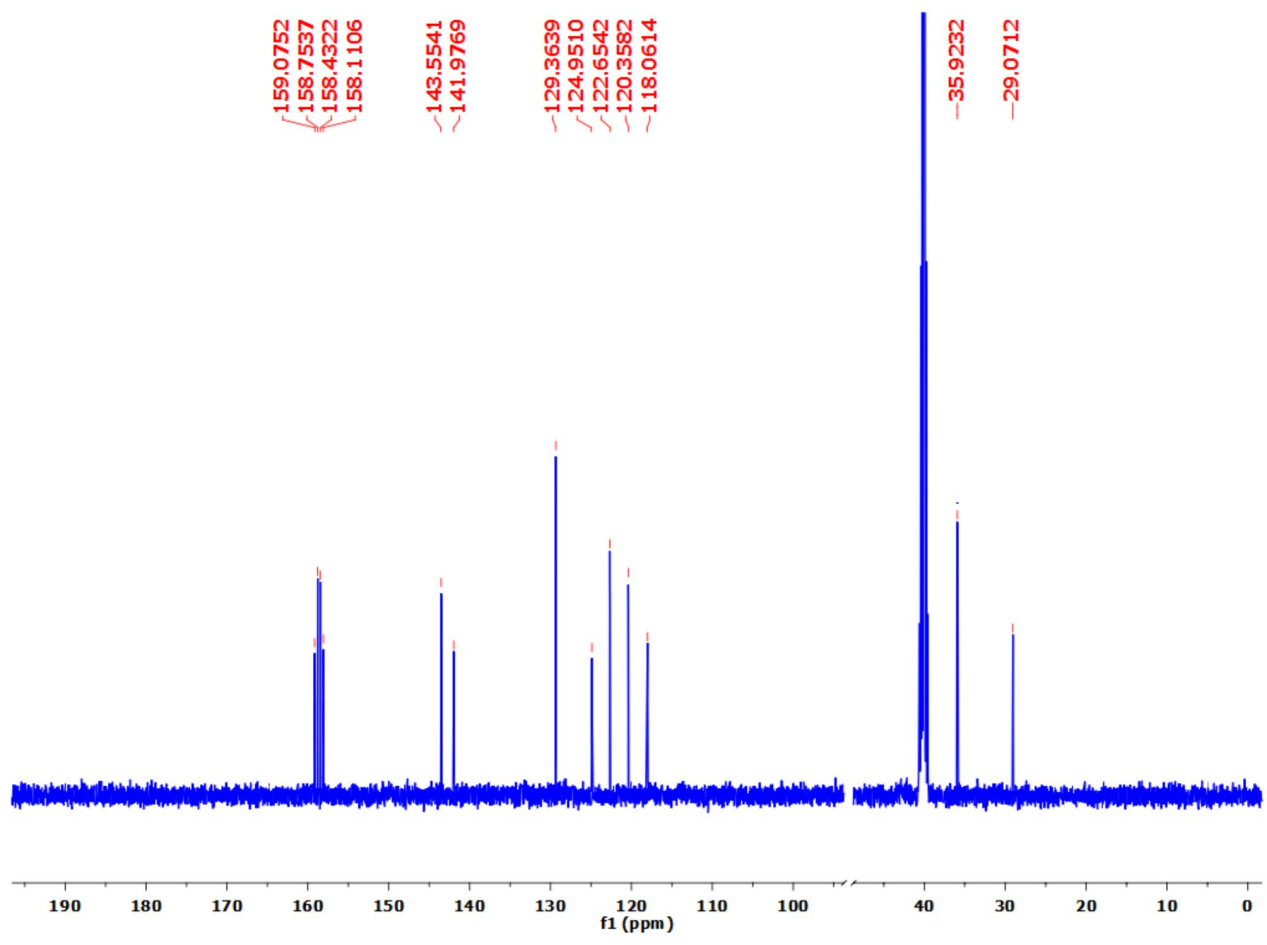
^13^C NMR spectrum of PBPBSDT.

**Fig. 8:**
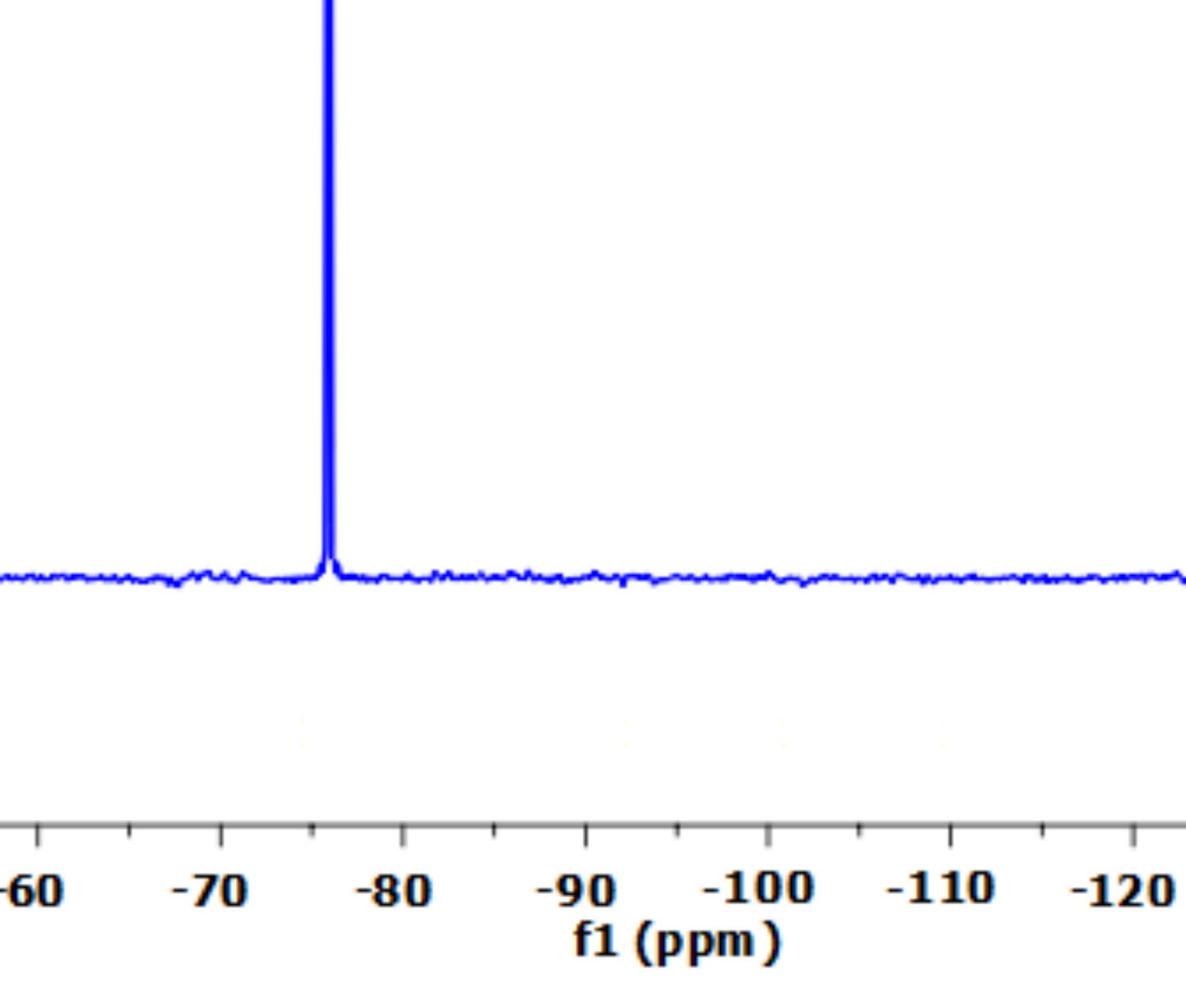
^19^F NMR spectrum of PBPBSDT.

The proton (^1^H), carbon (^13^C), and fluorine (^19^F) NMR spectra of PBPBSDT are shown in Figs. 6, 7 and 8, correspondingly. The data of these three spectra well defines the generation of PBPBSDT as follows: ^1^H NMR (500 MHz, DMSO-*d*_*6*_) δ (ppm): 9.96 (br., 2 H, OH), 9.11 (d, *J* = 8.1 Hz, 4 H, H_Ar_), 8.40 (d, *J* = 8.1 Hz, 4 H, H_Ar_), 2.59 (t, J = 9.9 Hz, 4 H, 2CH_2_), 1.94 (quintet, J = 9.9 Hz, 2 H, CH_2_) (Fig. 6); ^13^C NMR (125 MHz, DMSO-*d*_*6*_) δ (ppm): 159.07–158.11 (q, ^*2*^*J*_*C−F*_ = 40.2 Hz), 143.55, 141.97, 129.36, 124.95–118.06 (q, ^*1*^*J*_*C−F*_ = 287.1 Hz), 35.92, 29.07 (Fig. 7); ^19^F NMR (235 MHz, DMSO-d6) δ (ppm): -70.59 ppm (Fig. 8).

### Catalytic experiments

#### Producing dibenzo-chromeno-phenazine-diones catalyzed using PBPBSDT

In this section, how to optimize the protocol for creating C-N, C = N, C-C, C = C, C-O bonds during the production of dibenzo-chromeno-phenazine-dione derivatives under solvent-free conditions in a single operation via one-pot multicomponent domino reaction was monitored. In this regard, the reaction between 1 mmol benzene-1,2-diamine, 2 mmol 2-hydroxynaphthalene-1,4-dione, and 1 mmol 3-nitrobenzaldehyde was considered as a benchmark reaction to implement this idea. We first studied the progress of the model reaction in the presence of 5 mol% of each component of the PBPBSDT skeleton, and 1,3-n-propyl-bipyridinium bisulfonic acid-dichloride (Intermediate **I**), at a temperature of 80 ºC (Table 2, entries 1–4). The results showed that none of them alone can be a suitable catalyst for improving the reaction conditions (Time: 20 min, Yield: 25–67%). At 100 °C, no product was produced even after 60 min without PBPBSDT (Table 2, entry 5). Then, we investigated the reaction in the presence of 3 to 8 mol% of ionic liquid at a temperature of 40 to 70 °C (Table 3, entries 6–10). Table 2 showed, the best temperature for this synthetic conversion is 60 °C (Table 2, entry 6). It should be noted that by increasing the temperature to 70 °C, no significant difference in product yield was seen (Table 2, entry 9), but decreasing it to 40 °C caused the yield to drop to 73% (Table 2, entry 7). Accuracy in the results of entries 6, 8, and 10 indicated that 5 mol% PBPBSDT was sufficient to quickly carry out the reaction (Table 2, entry 6). Therefore, the conditions mentioned in entry 6 was considered as the optimal conditions. It is noteworthy that the attempt with PTSA and K_2_CO_3_ as a catalyst under optimal conditions was not successful; so that the corresponding products were obtained in 20 min with a yield of 48% and trace (Table 2, entries 11 and 12).

**Table 2:** Screening of reaction conditions for preparing **5a** in the absence of solvent.

Entry	Catalyst	Temp. (°C)	Catalyst amount (mol%)	Time (min)	Yield^a^ (%)
1	1,3-di(pyridin-4-yl)propane	80	5	20	25
2	Chlorosulfonic acid	80	5	20	29
3	Trifluoroacetic acid (TFA)	80	5	20	33
4	Intermediate I	80	5	20	67
5	–	100	–	60	NR^b^
6	PBPBSDT	60	5	10	97
7	PBPBSDT	40	5	10	73
8	PBPBSDT	60	8	10	98
9	PBPBSDT	70	5	10	98
10	PBPBSDT	60	3	10	70
11	PTSA (as an acid)^c^	60	5	20	48
12	K_2_CO_3_ (as a base)	60	5	20	Trace

^a^Isolated yield.

^b^ No reaction.

^c^*p*-Toluenesulfonic acid.

Next, the versatility and activity scope of PBPBSDT as a bi-functional ionic liquid catalyst was evaluated by reacting different aromatic aldehydes under optimal conditions (Table 3). All reactions were completed in 9 to 15 min and led to the formation of target molecules (Time: 9–15 min, Yield: 90–98%) in high yield. Therefore, it was confirmed that PBPBSDT is a suitable catalyst for this synthetic transformation and our idea for its design was reasonable.

**Table 3:** Synthesizing dibenzo-chromeno-phenazine-diones catalyzed via PBPBSDT.


Product	Ar	Time (min)	Yield (%)^a^	TON	TOF (min^− 1^)	M.p. (ºC) Found (Reported)
**1a**	4-O_2_N-C_6_H_4_	9	98	19.60	2.17	273–275 (274–275) ^35^
**2a**	3-O_2_N-C_6_H_4_	10	98	19.60	1.96	369–371 (368–370) ^36^
**3a**	2-O_2_N-C_6_H_4_	10	97	19.40	1.94	292–294 (293–295) ^37^
**4a**	3-CN-C_6_H_4_	10	97	19.40	1.94	287–289 (286–288) ^38^
**5a**	4-Cl-C_6_H_4_	10	98	19.60	1.96	324–326 (324–326) ^36^
**6a**	2-Cl-C_6_H_4_	12	94	18.80	1.56	334–336 (336–338) ^36^
**7a**	2,4-Cl_2_-C_6_H_3_	13	92	18.40	1.41	329–331 (330–332) ^36^
**8a**	4-Me-C_6_H_4_	12	93	18.60	1.55	333–335 (332–334) ^35^
**9a**	4-MeO-C_6_H_4_	13	94	18.80	1.44	339–341 (341–342) ^36^
**10a**	3,4-(MeO)_2_-C_6_H_3_	15	90	18.00	1.20	319–322 (320–321) ^38^
**11a**	4-OH-C_6_H_4_	13	91	18.20	1.40	299–301 (301–303) ^38^
**12a**	2-OH-5-NO_2_-C_6_H_4_	15	90	18.00	1.20	364–366 (365) ^38^

^a^Isolated yield.

**Fig. 9:**
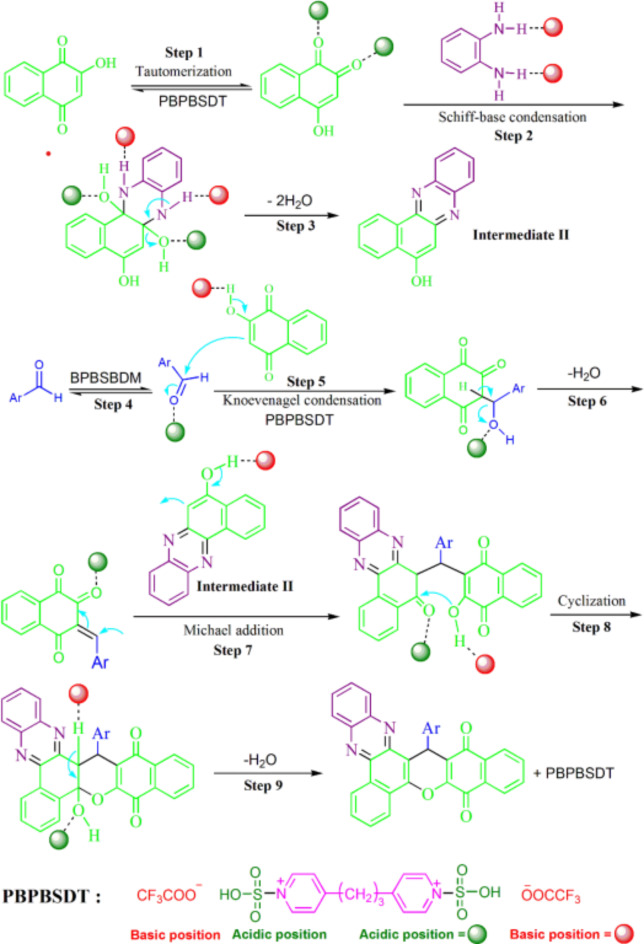
A proposed mechanism in the preparing dibenzo-chromeno-phenazine-diones.

PBPBSDT ionic liquid is a bi-functional catalyst due to having both acidic (–SO_3_H) and basic (CF_3_COOˉ) positions^8,11^. In Fig. 9, an interesting mechanism is shown with the help of its acidic and basic sites to produce dibenzo-chromeno-phenazine-diones. Previous literature supports the dual functionality of PBPBSDT^35,36,44,45^. The reaction mechanism includes 9 stages, and the ionic liquid helps to carry out the following processes: expediting the tautomerization reaction (step 1), activation of the nucleophiles (steps 2, 5, 7 and 8), activation of the electrophiles (steps 1, 4, 7 and 8), facilitating water elimination (steps 3, 6 and 9), and accelerating the cyclization step (step 8).

#### Catalyst recyclability

**Fig. 10:**
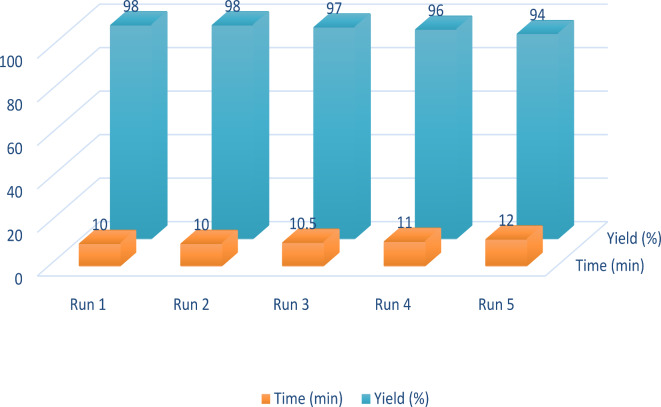
The results of five times of PBPBSDT recycling during the production of derivative **2a**.

The reproducibility (i.e., recyclability and reusability) of catalysts, especially their homogeneous types, is an essential advantage for commercial and industrial applications. Accordingly, after the completion of the reaction, the remainder was extracted by adding 20 mL of hot ethyl acetate to isolate PBPBSDT (because the catalyst, unlike the product, is insoluble in this solvent). The removed catalyst was triturated with a one-to-one mixture of ethanol and ethyl acetate and dried at 60 °C for next use. In Fig. 10, the results of five times PBPBSDT recycling during the production of derivative **2a** are displayed as a function of time and product percentage. The slight decline in the performance of our ionic liquid is due to its loss of 3–5% during the recovery process.

### Comparison

To appraise the current protocol against other reported approaches in the producing dibenzo-chromeno-phenazine-dione derivatives, the catalytic ability of the PBPBSDT was compared with some catalysts published in the literature, with the criterion of placing factors such as the reaction medium with or without solvent, reaction time, amount of catalyst used, and product yield. For doing this, the reaction of preparing product **2a** was considered as a model and the results were tabulated in Table 4. As the table shows, PBPBSDT is superior to the reported catalysts in most parameters mentioned. In addition, this ionic liquid catalyst is comparable in terms of low toxicity, easy isolation, and availability and low price of its parents.

**Table 4:** Evaluation of the catalytic performance of PBPBSDT with other available catalysts in the producing dibenzo-chromeno-phenazine-diones.

Catalyst	Conditions	Time (min)	Yield^a^ (%)	Ref.
PBPBSDT (5 mol%)	Solvent-free, 60 ºC	10	98	–
Zr-guanine-MCM-41 (0.30 mol%)	PEG-400, 100 ºC	120	90	^35^
*para*-Toluenesulfonic acid (20 mol%)	PEG-400, 80 ºC	120	91	^36^
Ni-Gly-isatin@boehmite (0.04 g)	PEG-400, 80 ºC	300	90	^37^
γ-Fe_2_O_3_@SiO_2_‐SCH_2_CO_2_H (0.03 g)	EtOH/H_2_O (1:1), 70 ºC	120	94	^38^
L-proline (20 mol%)	H_2_O, Microwave, 180 W, max. 70 °C	10	95	^39^

^a^Isolated yield.

## Conclusions

Concisely, we have presented 1,3-n-propyl-bipyridinium bisulfonic acid-ditrifluoroacetate (PBPBSDT), as a highly capable, homogeneous, and bi-functional ionic liquid in the preparing dibenzo-chromeno-phenazine-dione derivatives through the one-pot MCDR of 1 equivalent aryl aldehydes, 2 equivalents 2-hydroxynaphthalene-1,4-dione, and 1 equivalent benzene-1,2-diamine under the facile condition with high yields in short reaction times (solvent-free, 60 °C, 90–98%, 9–15 min). Then, in justifying its influential role as an acid and base duel in promoting the reaction, a suitable mechanism was demonstrated with the help of its acidic and basic position (i.e., –SO_3_H and CF_3_COOˉ). Appropriate reproducibility of the catalyst despite its homogeneous nature (5 times), operational simplicity, solvent-free conditions, and easy work-up are the other benefits of this methodology.

## Data Availability

The data that support the findings of this study are available on request from the corresponding author.
